# Upregulated Intrathecal Expression of VEGF-A and Long Lasting Global Upregulation of Proinflammatory Immune Mediators in Vaccine Breakthrough Tick-Borne Encephalitis

**DOI:** 10.3389/fcimb.2021.696337

**Published:** 2021-07-01

**Authors:** Miša Pavletič, Misa Korva, Nataša Knap, Petra Bogovič, Lara Lusa, Klemen Strle, Mirijam Nahtigal Klevišar, Tomaž Vovko, Janez Tomažič, Stanka Lotrič-Furlan, Franc Strle, Tatjana Avšič-Županc

**Affiliations:** ^1^ Laboratory for Diagnostic of Zoonoses and World Health Organization (WHO) Center, Institute of Microbiology and Immunology, Faculty of Medicine, University of Ljubljana, Ljubljana, Slovenia; ^2^ Department of Infectious Diseases, University Medical Center Ljubljana, Ljubljana, Slovenia; ^3^ Institute for Biostatistics and Medical Informatics, Faculty of Medicine, University of Ljubljana, Ljubljana, Slovenia; ^4^ Department of Mathematics, Faculty of Mathematics, Natural Sciences and Information Technologies, University of Primorska, Koper, Slovenia; ^5^ Division of Infectious Diseases, Microbial Pathogenesis and Immunology Laboratory, Wadsworth Center, New York State (NYS) Department of Health, Albany, NY, United States

**Keywords:** tick-borne encephalitis, vaccine breakthrough, cytokines, chemokines, proinflammatory response, VEGF

## Abstract

Although anti-TBE vaccines are highly effective, vaccine breakthrough (VBT) cases have been reported. With increasing evidence for immune system involvement in TBE pathogenesis, we characterized the immune mediators reflecting innate and adaptive T and B cell responses in neurological and convalescent phase in VBT TBE patients. At the beginning of the neurological phase, VBT patients have significantly higher serum levels of several innate and adaptive inflammatory cytokines compared to healthy donors, reflecting a global inflammatory state. The majority of cytokines, particularly those associated with innate and Th1 responses, are highly concentrated in CSF and positively correlate with intrathecal immune cell counts, demonstrating the localization of Th1 and proinflammatory responses in CNS, the site of disease in TBE. Interestingly, compared to unvaccinated TBE patients, VBT TBE patients have significantly higher CSF levels of VEGF-A and IFN-β and higher systemic levels of neutrophil chemoattractants IL-8/CXCL8 and GROα/CXCL1 on admission. Moreover, serum levels of IL-8/CXCL8 and GROα/CXCL1 remain elevated for two months after the onset of neurological symptoms, indicating a prolonged systemic immune activation in VBT patients. These findings provide the first insights into the type of immune responses and their dynamics during TBE in VBT patients. An observed systemic upregulation of neutrophil derived inflammation in acute and convalescent phase of TBE together with highly expressed VEGF-A could contribute to BBB disruption that facilitates the entry of immune cells and supports the intrathecal localization of Th1 responses observed in VBT patients.

## Introduction

Tick-borne encephalitis (TBE), one of the most common viral infection of central nervous system in Europe and Asia, is caused by tick-borne encephalitis virus (TBEV) that belongs to the genus *Flavivirus*, family *Flaviviridae*. The main route of infection with European subtype of TBEV (Eu-TBEV) is by infected *Ixodes ricinus* tick bites, however the infection is also associated with consumption of unpasteurized milk/dairy products from TBEV infected livestock (Lindquist and Vapalahti, 2008; Hudopisk et al., 2013). Infections with the Eu-TBEV are mainly asymptomatic, but in the majority of clinically manifested infections the disease has a bi-phasic course. After the first phase, which is characterized by fever and nonspecific symptoms, and a short interval without any signs of infection, patients develop neurological symptoms that can vary from mild (meningitis) to severe (meningoencephalitis, meningoencephalomyelitis). Rarely, patients may directly develop neurological symptoms without preceding febrile illness, or they may present with an initial phase of TBE without subsequent CNS involvement (Lotric-Furlan et al., 2002; Kaiser, 2008; Bogovic et al., 2010).

The disease course and outcome is determined by the intricate virus-host interactions at several steps of infection. An important event in the pathogenesis of TBE is the breakdown of blood-brain barrier (BBB) that enables the progression of central nervous system (CNS) infection and infiltration of immune cells into the brain. Increased permeability of BBB, however, is a consequence rather than a prerequisite for CNS infection, most likely caused by the release of proinflammatory mediators from the initial infection of brain tissue (Song and Pachter, 2004; Ruzek et al., 2011; Palus et al., 2014; Palus et al., 2015). Although a robust immune response is crucial for viral clearance and disease prevention in early stages of infection (King et al., 2007; Lindqvist et al., 2016), there is increasing evidence of a direct immune system involvement in neuropathological manifestations of TBE (Lepej et al., 2007; Gunther et al., 2011; Kang et al., 2013; Grygorczuk et al., 2017; Grygorczuk et al., 2018; Bogovic et al., 2019). In patients with TBE, levels of Th1 cytokines as well as leucocyte chemoattractants are highly concentrated in CSF at the beginning of neurological phase of the disease (Lepej et al., 2007; Grygorczuk et al., 2017; Bogovic et al., 2019). Additionally, upregulated expression of Th17 cytokines and neutrophil chemoattractants in CSF of TBE patients support the likelihood of neutrophil derived inflammation in the CNS (Zajkowska et al., 2011; Grygorczuk et al., 2018). In contrast, elevated CSF levels of anti-inflammatory mediator IL-10, as well as IFN-β and IFN-λ3 were detected in patients with milder clinical presentation, suggesting their protective role in TBE pathogenesis (Gunther et al., 2011; Grygorczuk et al., 2015). The efficacy of immune response against TBEV infection is also influenced by host genetic factors as severe manifestation of TBE were associated with polymorphisms in *TLR3* (Kindberg et al., 2011; Barkhash et al., 2013; Mickiene et al., 2014), *CCR5* (Mickiene et al., 2014),*CD209* (Barkhash et al., 2012), and *IL10* genes (Barkhash et al., 2016).

Currently the most important protective measure against infection with TBEV is regular vaccination that successfully promotes the formation of neutralizing antibodies in 98-99.5% of vaccinees (Heinz et al., 2013). Two prophylactic vaccines against TBE available in Europe, FSME IMMUN (Pfizer Luxembourg SARL, Luxembourg) and Encepur (GSK Vaccines GmbH, Germany), have highly homologous antigenic components and trigger the formation of cross-reactive and neutralizing antibodies that provide protection against all Eu-TBEV subtypes (Orlinger et al., 2011; McAuley et al., 2017). To maintain the protective level of antibodies against TBEV regular booster doses are recommended. Due to impairment of immune functions in older individuals, shorter booster intervals are suggested for those over 50 (60) years old (Stiasny et al., 2012). Even with high reported TBE incidence rate in Slovenia (3.0-15.0 TBE cases/100.000 inhabitants in the period from 2008 to 2018), National Institute of Public Health estimated that in 2014 only approximately 16% of the population was vaccinated with at least one dose and around 7% of the population was vaccinated completely and regularly^1^.

Despite highly immunogenic and effective vaccine against TBE, vaccine breakthroughs (VBT) have been reported in several European countries, including Slovenia (Bender et al., 2004; Kleiter et al., 2007; Stiasny et al., 2009; Andersson et al., 2010; Zlamy et al., 2016; Lotric-Furlan et al., 2017). Vaccine failures are probably very rare but can develop not only after incomplete but also after complete primary immunization (i.e., completion of 3-dose vaccination) and usually occur in elderly patients (Kleiter et al., 2007; Stiasny et al., 2009; Andersson et al., 2010; Grgic-Vitek et al., 2010; Lotric-Furlan et al., 2017). However, there have also been reports of TBE vaccine failures in children (Stiasny et al., 2009; Zlamy et al., 2016). In contrast to unvaccinated TBE patients who initially develop high serum levels of IgM antibodies that are followed by low specific IgG at the beginning of neurologic involvement, neurological symptoms in VBT patients are accompanied by markedly elevated levels of specific and neutralizing IgG antibodies with high avidity to TBEV and by the absence or low levels of IgM antibodies indicating an anamnestic immune response (Stiasny et al., 2009; Lotric-Furlan et al., 2017).

Although several studies have investigated the characteristics of antibody response in VBT patients, information on other immunological features are lacking. In this study, we performed an in-depth analysis of cytokines and chemokines associated with innate and adaptive (T and B cell) immune responses in TBE vaccine breakthrough patients. These data provide the first insights into the involvement of cellular immune responses in the pathogenesis of TBE in patients previously vaccinated against this disease.

## Materials and Methods

### Study Group Characteristics

The study included 41 TBE patients (24 males, 59% and 17 females, 41%; median age 61 years; IQR 46-65 years) who had received at least two doses of TBE vaccine prior to TBE and 81 age- and gender-matched unvaccinated patients (47 males, 58% and 34 females, 42%; median age was 58 years, IQR 47-66 years) diagnosed with TBE in the same year. All TBE patients enrolled in our study were diagnosed at the Department of Infectious Diseases, UMC Ljubljana, Slovenia, from January 2003 to December 2018. The diagnosis was based on clinical signs (symptoms of meningitis/meningoencephalitis), elevated CSF leucocyte count (>5 x 10^6 cells L-1), and the presence of serum IgM and IgG antibodies to TBEV on admission to the hospital. For patients with breakthrough TBE, intrathecal synthesis of specific IgM and/or IgG antibodies was also evaluated. The control group consisted of 42 healthy age- and gender-matched individuals (20 males, 47% and 22 females, 53%; median age 56.5 years, IQR 46.3-56.5).

### Sample Collection

For both groups of TBE patients, matched serum and CSF samples were obtained at the beginning of the neurological phase on admission to the hospital. Additional serum samples were drawn on follow-up examinations; from 18/41 VBT patients two to three weeks and from 24/41 VBT patients one to two months after the onset of neurological symptoms. Patients had not received any immunotherapy prior to sample collection. Serum samples of healthy individuals (control group) were collected in December of 2018. All samples were aliquoted and stored at -80°C from 6 months to 15 years until further analysis.

### Ethics Statement

The study was carried out in concordance with the Declaration of Helsinki and was approved by the National Medical Ethic Committee (approval number 131/06/13, 0120-188/2018/6, 81/12/2013, 0120-188/2018/6, and 0120-564/2018/13).

### Laboratory Techniques

Peripheral blood and CSF leukocyte counts were assessed with standard laboratory techniques. Anti-TBEV IgM and IgG antibodies in serum and CSF were determined using Enzygnost^®^ Anti-TBE virus (IgM, IgG) test (SiemensGmbH, Marburg, Germany) according to the manufacturer’s instructions.

Concentrations of 35 cytokines and chemokines associated with innate and adaptive immune responses were measured in paired serum and CSF samples obtained from VBT patients and unvaccinated TBE patients at the first visit, in serum samples of VBT patients drawn at follow-up time points (two to three weeks and one to two months after the onset of neurological symptoms) as well as in serum samples of healthy controls (n=42). The concentrations of immune mediators were measured using Human Cytokine/Chemokine Magnetic Bead Panel, Human Th17 Magnetic Bead Panel, Human Cytokine/Chemokine Panel II, Human Cytokine/Chemokine Magnetic Bead Panel III and Human Cytokine/Chemokine Magnetic Bead Panel IV (Millipore, Germany), and APRIL Human ProcartaPlex Simplex Kit (ThermoFisher Scientific, Waltham, MA) for the Luminex platform on MAGPIX instrument (Luminex, Austin, TX) according to the manufacturer’s instructions. The immunoassays detect interleukin 1β (IL-1β), IL-1 receptor agonist (IL-1RA), interleukin 2 (IL-2), interleukin 4 (IL-4), interleukin 6 (IL-6), interleukin 8 (IL-8), interleukin 10 (IL-10), interleukin 12 [IL-12 (p40)], interleukin 15 (IL-15), interferon alpha (IFN-α), interferon beta (IFN-β), interferon gamma (IFN-γ), interferon lambda-3 (IFN-λ3/IL-28B), tumor necrosis factor alpha (TNF-α), granulocyte macrophage colony stimulating factor (GM-CSF), GROα (CXCL1), inducible protein 10 (IP-10/CXCL10), macrophage chemotactic protein 1 (MCP-1/CCL2), macrophage inflammatory protein 1α (MIP-1α/CCL3), vascular endothelial growth factor (VEGF-A), interleukin 17A (IL-17A), interleukin 17F (IL-17F), interleukin 21 (IL-21), interleukin 22 (IL-22), interleukin 23 (IL-23), interleukin 25 (IL-25), interleukin 27 (IL-27), stromal cell-derived factor 1 (SDF-1α+β/CXCL12), B cell-attracting chemokine 1 (BCA-1/CXCL13), monokine induced by gamma interferon (MIG/CXCL9), interferon-inducible T-cell alpha chemoattractant (I-TAC/CXCL11), macrophage inflammatory protein 3β (MIP-3β/CXCL19), B-cell activating factor (BAFF) and a proliferation-inducing ligand (APRIL). Serum and CSF samples were diluted 1:5 with Assay Buffer (Millipore, Germany) provided in the multiplex assay kits. All measurements with a single panel were carried out on the same day in one complete experiment to minimize inter-assay variation. Data analysis was performed simultaneously for all plates in a single panel using Milliplex Analyst 5.1 Software. All values outside the upper and lower end of the standard curve were considered as maximum or minimum values, respectively.

### Statistical Analysis

The comparison of cytokine and chemokine concentrations between groups were analyzed with R statistical language (R), version 3.5.0. Mann-Whitney test was used for two-group comparisons and Kruskal-Wallis rank sum test for multiple groups. The univariate P-values of obtained for each research question (as presented in tables and figures) were adjusted for multiple testing using a multivariate permutation method (Westfall et al., 2013), using 1000 permutations. The correlation between cytokine/chemokine levels and IgG antibodies in serum or CSF was assessed by Spearman’s correlation method in GraphPad Prism, version 7 (GraphPad Software, San Diego, CA). The same approach was used for the evaluation of correlation between cytokine/chemokine levels and leukocyte counts in serum and CSF, respectively. Adjusted P values <=0.05 were considered statistically significant.

## Results

To characterize innate and adaptive immune responses in patients with VBT TBE, we measured concentrations of 35 soluble immune mediators in paired serum and CSF samples of 41 VBT patients taken on admission, and in convalescent serum samples obtained at follow-up examinations two to three weeks and one to two months after discharge. Paired serum and CSF samples from the beginning of meningoencephalitic phase of TBE were obtained simultaneously within the period of 6 hours. Furthermore, we compared the levels of immune mediators in serum and CSF of VBT patients with the corresponding findings obtained in serum and CSF samples of 81 age- and gender-matched unvaccinated patients with TBE, as well as with serum levels of immune mediators in 42 age- or gender-matched healthy controls. To further investigate the role of cytokines and chemokines in VBT patients, we analyzed the relation between concentrations of immune mediators and levels of anti-TBEV IgG antibodies and leukocyte counts in paired serum and CSF samples obtained on admission to the hospital, as well with anti-TBEV IgG antibodies in serum samples taken in convalescent phase of the disease. Basic clinical and laboratory characteristics of VBT patients are presented in **Table 1**.

**Table 1:** Clinical and laboratory data on 41 VBT patients enrolled in the study.

Characteristics		Number (%) or Median (IQR)
**Vaccination status**	Complete	29 (70.7%)
	Incomplete	12 (29.3%)
**Underlying illnesses**		21 (55.3%)^a^
**Monophasic course of illness**		37 (90.2%)
**IgG Antibody concentration (U/ml)**	Serum	341 (156-575)
	CSF	333 (143.5-575)^b^
**Blood leukocyte count (x10^9/L)**		10.2 (8.9-13.1)^c^
	Neutrophils (x10^9/L)	8.2 (6.6-10.6)^d^
	Lymphocytes (x10^9/L)	1.0 (0.7-1.4)^d^
	Monocytes (x10^9/L)	0.1 (0.04-0.1)^d^
**CSF leukocyte count (x10^6/L)**		130.5 (61.5-356.0)^c^
	Neutrophils (x10^6/L)	27.0 (11.0-73.5)^c^
	Lymphocytes (x10^6/L)	66.5 (32.0-165.3)^c^
	Monocytes (x10^6/L)	4 (0.25-9.8)^c^

Data are shown as median (IQR) or number (%). ^a^Data available for 39 VBT patients. ^b^Data available for 35 VBT patients. ^c^Data available for 38 VBT patients. ^d^Data available for 37 VBT patients. IQR, interquartile range; CSF, cerebrospinal fluid.

### Cytokine and Chemokine Concentrations in Acute Serum Samples of Patients With VBT TBE In Comparison to Healthy Controls

**Figure 1:**
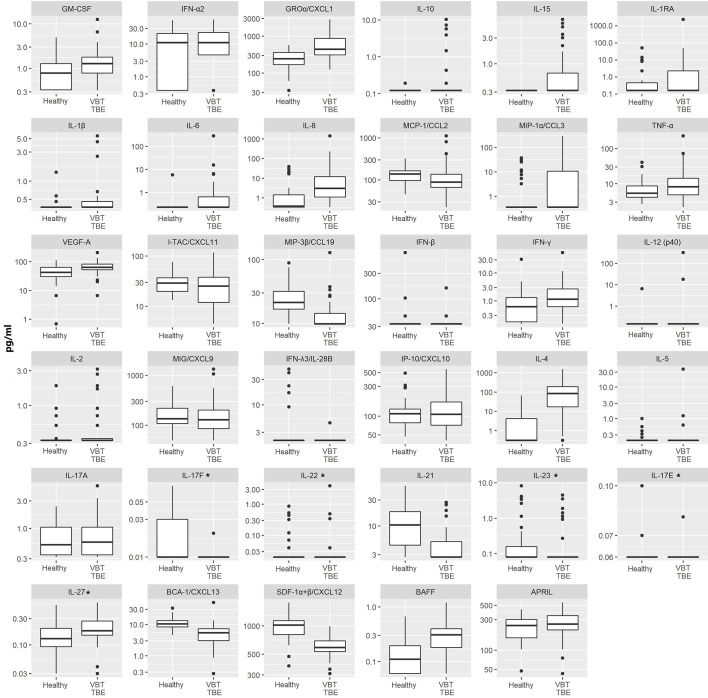
Serum concentrations of 35 immune mediators in VBT patients (VBT; n = 41) and age and gender matched healthy individuals (Healthy; n = 42). For VBT patients, cytokine and chemokine concentrations measured in serum samples obtained at the onset of neurological symptoms are presented. Concentrations of immune mediators are in pg/ml, except those, marked with an asterisk are in ng/ml. The box and whisker plots show the median (vertical line), quartiles (box) and the whiskers, which extend to the most extreme data point which is no more than 1.5 times the interquartile range from the box; the data points outside of this range are plotted individually. Innate immune mediators: GM-CSF, IFN-α2, GROα/CXCL1, IL-10, IL-15, IL-1RA, IL-1β, IL-6, IL-8, MCP-1/CCL2, MIP-1α/CCL3, TNF-α, VEGF-A, MIP-3β/CCL19, IFN-β; Th1 immune mediators: IFN-γ, IL-12 (p40), IL-2, MIG/CXCL9, IFN-λ3/IL-28B, IP-10/CXCL10, I-TAC/CXCL11; Th2 immune mediators: IL-4, IL-5; Th17 immune mediators: IL-17A, IL-17F, IL-22, IL-21, IL-23, IL-17E, IL-27; B cell immune mediators: BCA-1/CXCL13, SDF-1α+β/CXCL12, BAFF, APRIL.

On admission, VBT patients had significantly higher serum levels of several innate proinflammatory cytokines and chemokines, including VEGF-A, GROα/CXCL1, IL-6, IL-8, and IL-15 as well as one Th2 mediator (IL-4) and one B cell mediator (BAFF) compared to healthy controls (p<0.05). A similar trend was observed for one Th1 cytokine, IFN-γ. In contrast, serum levels of one innate immune chemokine MIP-3β/CCL3, two Th17 (IL-17F and IL-21) cytokines, and two B cell chemoattractants (SDF-1α+β/CXCL12 and BCA-1/CXCL13) were significantly lower (p<0.05) in VBT patients compared to healthy controls (**Table 2** and **Figure 1**).

**Table 2:** Serum concentrations of cytokines/chemokines obtained in patients during meningoencephalic phase of TBE and in healthy controls.

Cytokine/Chemokine	Concentrations (pg/ml) Median (IQR)		p Value	p Value Adjusted
	VBT TBE	Healthy		
**Innate**				
GM-CSF	1.28 (0.79-1.79)	0.79 (0.33-1.28)	**0.017**	0.357
IFN-α2	11.05 (4.63-22.4)	11.05 (0.38-21.04)	0.102	0.953
GROα/CXCL1	**441.21 (304.58-867.17)**	245.41 (169.87-361.36)	**<0.001**	**0.001**
IL-10	0.12 (0.12-0.12)	0.12 (0.12-0.12)	**0.003**	0.062
IL-15^a^	**0.32 (0.32-0.67)**	0.32 (0.32-0.32)	**<0.001**	**0.001**
IL-1RA	0.16 (0.16-2.27)	0.16 (0.16-0.52)	**0.030**	0.538
IL-1β	0.37 (0.37-0.46)	0.37 (0.37-0.37)	**0.016**	0.346
IL-6a	**0.23 (0.23-0.64)**	0.23 (0.23-0.23)	**<0.001**	**0.011**
IL-8	**3.05 (1.05-12.05)**	0.38 (0.34-1.42)	**<0.001**	**0.001**
MCP-1/CCL2	88.44 (65.38-136.72)	138.53 (92.44-165.23)	**0.010**	0.230
MIP-1α/CCL3	0.36 (0.36-10.71)	0.36 (0.36-0.36)	**0.019**	0.400
TNF-α	8.27 (4.77-14.3)	5.42 (4.11-8.66)	**0.048**	0.735
VEGF-A	**63.69 (49.01-82.9)**	42.29 (27.84-63.69)	**0.002**	**0.047**
MIP-3β/CCL19	9.89 (9.89-14.39)	**21.30 (16.27-31.82)**	**<0.001**	**0.001**
IFN-β	33.84 (33.84-33.84)	33.84 (33.84-33.84)	0.692	1.000
**Th1**				
IFN-γ	1.14 (0.62-2.69)	0.62 (0.15-1.31)	**0.004**	0.108
IL-12 (p40)	0.15 (0.15-0.15)	0.15 (0.15-0.15)	0.535	1.000
IL-2	0.33 (0.33-0.35)	0.33 (0.33-0.33)	0.132	0.979
MIG/CXCL9	128.70 (82.059-200.77)	134.16 (103.49-219.46)	0.458	1.000
IFN-λ3/IL-28B	2.11 (2.11-2.11)	2.11 (2.11-2.11)	**0.007**	0.176
IP-10/CXCL10	107.20 (69.31-169.17)	109.27 (75.21-130.25)	0.757	1.000
I-TAC/CXCL11	25.20 (12.01-38.07)	28.98 (19.60-37.12)	0.316	1.000
**Th2**				
IL-4	**83.72 (15.495-188.34)**	0.31 (0.31-4.35)	**<0.001**	**0.001**
IL-5	0.20 (0.20-0.20)	0.20 (0.20-0.20)	0.563	1.000
**Th17**				
IL-17A	0.58 (0.35-1.05)	0.525 (0.35-1.05)	0.582	1.000
IL-17F^a^	10 (10-10)	**10 (10-30)**	**<0.001**	**0.001**
IL-22	20 (20-20)	20 (20-20)	0.258	1.000
IL-21	2.72 (2.72-5.21)	**10.435 (4.33-18.40)**	**<.001**	**0.001**
IL-23	80 (80-80)	80 (80-160)	0.389	1.000
IL-17E	60 (60-60)	60 (60-60)	0.332	1.000
IL-27	180 (140-270)	130 (90-200)	**0.014**	0.292
**B cell**				
BCA-1/CXCL13	5.44 (2.41-7.31)	**10.465 (8.28-13.17)**	**<0.001**	**0.001**
SDF-1α+β/CXCL12	587.59 (530.27-694.88)	**1021 (799.10-1138.25)**	**<0.001**	**0.001**
BAFF	**0.31 (0.18-0.4)**	0.11 (0.06-0.20)	**<0.001**	**0.001**
APRIL	254.73 (206.41-346.39)	242.77 (155.98-301.30)	0.297	1.000

a Medians and IQRs are the same but the other data (in the 1st and 4th quartiles) are not. Significantly higher concentrations of cytokines in each group and the corresponding p values are shown in bold.

Thus, at the onset of neurological symptoms, VBT TBE patients present with systemic upregulation of innate inflammatory immune mediators, with particularly increased neutrophil chemoattractants in serum compared to healthy donors. Additionally, a similar trend was observed for one Th1 immune mediator IFN-γ, while the expression of cytokines related to Th2 and B cell responses, were more heterogenous. In contrast, at the onset of neurological symptoms in VBT TBE patients, Th17 mediators in serum were lower compared to healthy controls, however, a low variability in concentration of these immune mediators was observed for VBT TBE patients. Low variability and observed low concentrations of some immune mediators could be an artifact from degradation due to long-term storage.

### Comparison of Cytokine and Chemokine Concentrations in Serum and CSF of Patients With VBT TBE

**Figure 2:**
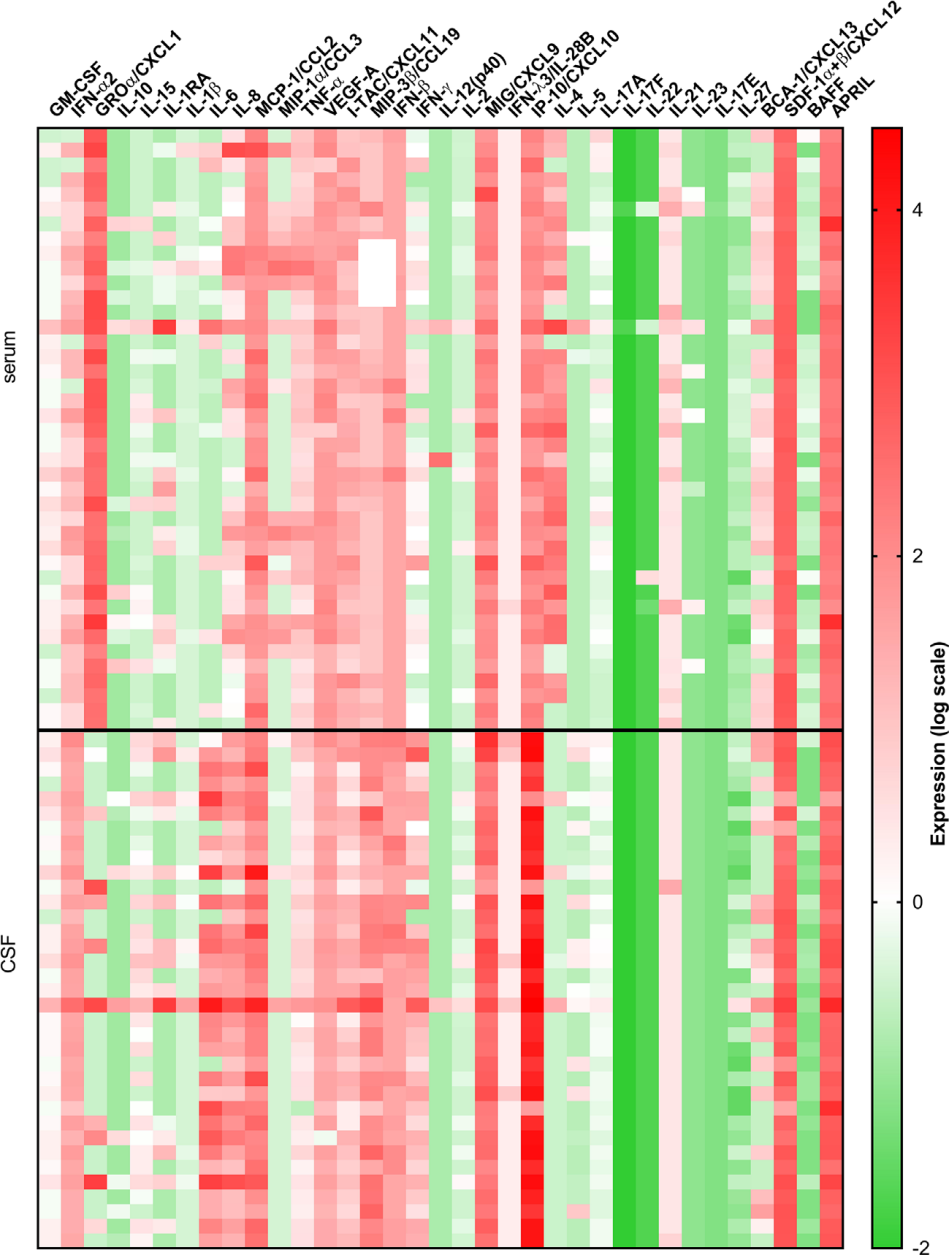
A heatmap illustrating concentrations of 35 cytokines and chemokines measured in serum and CSF of 41 VBT patients. Cytokine concentrations are visually presented on a log10 scale with red and green indicating high and low expression, respectively (refer to color bar).

Comparison between serum and CSF concentrations of immune mediators revealed differential localization of many cytokines and chemokines. Whereas the levels of several innate mediators (IFN-α2, IFN-β, IL-6, IL-8, IL-15, MCP-1/CCL-2, and MIP-3β/CCL19) were concentrated in CSF, others (GROα/CXCL1, TNF-α, and VEGF-A) were higher in serum. Similarly, Th2 cytokine IL-5, and B cell cytokine APRIL were higher in CSF, whereas IL-4 and BAFF were higher in serum. In contrast, Th1 associated mediators including IFN-γ, and IFN-γ-inducible chemokines MIG/CXCL9, and IP-10/CXCL10 were markedly elevated in CSF, whereas and I-TAC/CXCL11 was higher in serum (**Table 3** and **Figure 2**).

**Table 3:** Concentrations of cytokines/chemokines in serum and cerebrospinal fluid (CSF) obtained simultaneously during meningoencephalic phase of tick-borne encephalitis (TBE) in patients with vaccine breakthrough disease.

Cytokine/Chemokine	Concentrations (pg/ml) Median (IQR)		p Value	p Value Adjusted
	Serum	CSF		
**Innate**				
GM-CSF	1.28 (0.79-1.79)	1.28 (0.79-1.79)	0.232	0.986
IFN-α2	11.05 (4.63-22.4)	**41.25 (32.3-47.49)**	**<0.001**	**0.001**
GROα/CXCL1	**441.21 (304.58-867.17)**	0.30 (0.30-4.73)	**<0.001**	**0.001**
IL-10	0.12 (0.12-0.12)	0.12 (0.12-0.12)	0.779	1.000
IL-15	0.32 (0.32-0.67)	**2.05 (0.67-3.86)**	**<0.001**	**0.001**
IL-1RA	0.16 (0.16-2.27)	0.16 (0.16-6.15)	0.065	0.675
IL-1β	0.37 (0.37-0.46)	0.37 (0.37-0.37)	0.124	0.888
IL-6	0.23 (0.23-0.64)	**96.84 (25.10-271-92)**	**<0.001**	**0.001**
IL-8	3.05 (1.05-12.05)	**42.69 (14.00-83.39)**	**<0.001**	**0.007**
MCP-1/CCL2	88.44 (65.38-136.72)	**180.01 (92.77-432.31**)	**<0.001**	**0.001**
MIP-1α/CCL3	0.36 (0.36-10.71)	0.36 (0.36-0.36)	**0.005**	0.078
TNF-α	**8.27 (4.77-14.3)**	3.39 (2.16-4.59)	**<0.001**	**0.004**
VEGF-A	**63.69 (49.01-82.9)**	38.58 (25.63-45.78)	**0.001**	**0.009**
MIP-3β/CCL19	9.89 (9.89-14.39)	**125.15 (39.50-192.97)**	**<0.001**	**0.001**
IFN-β	33.84 (33.84-33.84)	**47.33 (33.84-103.04)**	**0.002**	**0.038**
**Th1**				
IFN-γ	1.14 (0.62-2.69)	**16.72 (7.86-37.60)**	**<0.001**	**0.001**
IL-12 (p40)	0.15 (0.15-0.15)	0.15 (0.15-0.15)	1.000	1.000
IL-2	0.33 (0.33-0.35)	0.35 (0.33-0.72)	0.106	0.850
MIG/CXCL9	128.70 (82.059-200.77)	**348.39 (242.46-618.28)**	**<0.001**	**0.001**
IFN-λ3/IL-28B	2.11 (2.11-2.11)	2.11 (2.11-2.11)	0.052	0.607
IP-10/CXCL10	107.20 (69.31-169.17)	**8056 (3886-15586)**	**<0.001**	**0.001**
I-TAC/CXCL11	**25.20 (12.01-38.07)**	12.01 (4.51-22.11)	**0.001**	**0.024**
**Th2**				
IL-4	**83.72 (15.495-188.34)**	0.31 (0.31-0.31)	**<0.001**	**0.001**
IL-5	0.20 (0.20-0.20)	**0.25 (0.20-1.39)**	**0.001**	**0.011**
**Th17**				
IL-17A	0.58 (0.35-1.05)	0.70 (0.47-0.81)	0.155	0.937
IL-17F	10 (10-10)	10 (10-10)	1.000	1.000
IL-22	20 (20-20)	20 (20-20)	0.181	0.970
IL-21	2.72 (2.72-5.21)	2.72 (2.72-2.72)	**0.012**	0.179
IL-23	80 (80-80)	80 (80-80)	**0.036**	0.474
IL-17E	60 (60-60)	60 (60-60)	1.000	1.000
IL-27	180 (140-270)	120 (60-220)	0.096	0.829
**B cell**				
BCA-1/CXCL13	5.44 (2.41-7.31)	1.78 (0.27-13.47)	0.285	0.998
SDF-1α+β/CXCL12	587.59 (530.27-694.88)	655.36 (410.61-907.95)	0.777	1.000
BAFF	**0.31 (0.18-0.4)**	0.06 (0.06-0.11)	**<0.001**	**0.001**
APRIL	254.73 (206.41-346.39)	**614.335 (432.92-801.10)**	**<0.001**	**0.001**

Significantly higher levels of cytokines/chemokines comparing the two compartments and the corresponding p values are shown in bold.

Thus, at the onset of neurological symptoms in VBT patients, the majority of inflammatory immune mediators, particularly those associated with innate and adaptive Th1 immune responses, were upregulated in CSF, suggesting the formation of intrathecal inflammatory focus. However, levels of Th2, Th17, and B cell cytokines were altered without a clear trend, indicating that these cellular immune responses are occurring in both compartments.

### Cytokine and Chemokine Concentrations in Acute Serum and CSF Samples of Patients With VBT TBE Compared to Unvaccinated TBE Patients

**Figure 3:**
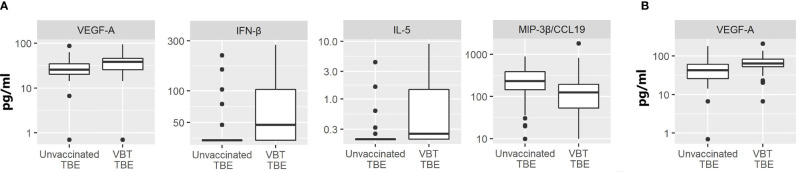
The concentrations of immune mediators that significantly differed in **(A)** CSF or **(B)** serum samples between VBT TBE patients (VBT TBE; n=41) and in age and gender matched unvaccinated TBE patients (Unvaccinated TBE; n=81) obtained at the onset of neurological symptoms.

In comparison to unvaccinated TBE patients, VBT patients had significantly higher CSF levels of VEGF-A, IFN-β and Th2 cytokine IL-5 (p<0.05), but lower concentration of MIP-3β/CCL19 (p<0.05) (**Figure 3A**).

The levels of immune mediators in serum did not differ significantly between VBT and previously unvaccinated patient with TBE, with the exception that VEGF-A concentration was higher in patients with VBT disease (**Figure 3B**). As VEGF-A has been implicated in degradation of extracellular matrix proteins through MMP-9, it is possible that its higher levels in VBT patients could be contributing to greater vascular permeability and more severe disease course. Additionally, two neutrophil chemoattractants, GROα/CXCL1 and IL-8, showed a tendency for higher expression in sera of VBT patients, possibly supporting increased BBB permeability and therefore facilitating the formation of intrathecal inflammatory focus. However, the observed difference was not significant (data not shown).

### Cytokine and Chemokine Concentrations in Relation to Vaccination Status, Age, and Gender

To evaluate the possible effect of age, gender or vaccination status of VBT TBE patients on their immune response, we compared serum and CSF concentrations of immune mediators between groups. Serum and CSF cytokine and chemokine levels did not differ between VBT patients with complete (3 doses of TBE vaccine) and incomplete vaccination (2 doses of vaccine) (**Supplementary Table 1**) nor between genders (**Supplementary Table 2**) and did not correlate with age (**Supplementary Table 3**).

### Longitudinal Analyses of Cytokine and Chemokine Levels in Serum of VBT Patients

**Figure 4:**
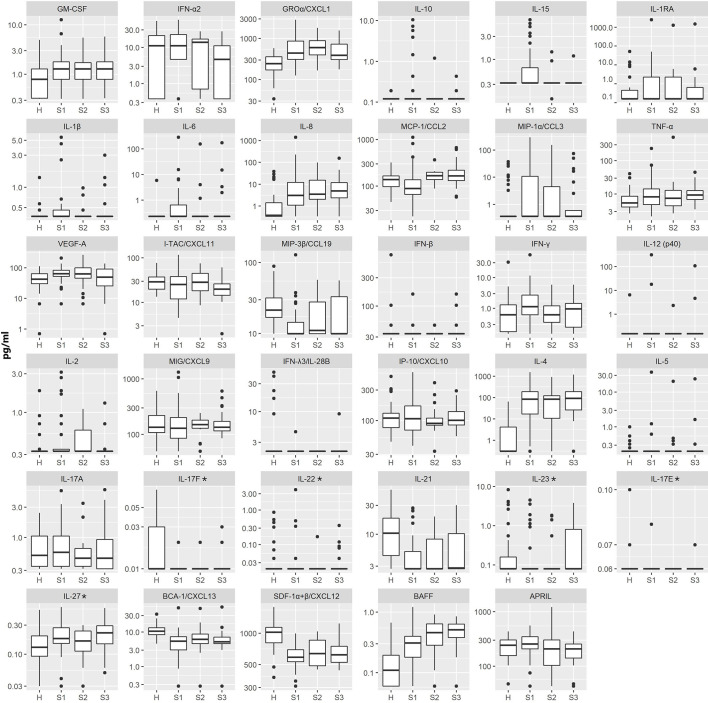
The concentrations of 35 immune mediators in serum samples of healthy individuals (H) and VBT TBE patients obtained on admission to the hospital (S1; n = 41), two to three weeks later (S2; n = 18) and one to two months (S3; n = 24) after the onset of neurological symptoms. Concentrations of immune mediators are in pg/ml, except those, marked with an asterisk are in ng/ml. Innate immune mediators: GM-CSF, IFN-α2, GROα/CXCL1, IL-10, IL-15, IL-1RA, IL-1β, IL-6, IL-8, MCP-1/CCL2, MIP-1α/CCL3, TNF-α, VEGF-A, MIP-3β/CCL19, IFN-β; Th1 immune mediators: IFN-γ, IL-12 (p40), IL-2, MIG/CXCL9, IFN-λ3/IL-28B, IP-10/CXCL10, I-TAC/CXCL11; Th2 immune mediators: IL-4, IL-5; Th17 immune mediators: IL-17A, IL-17F, IL-22, IL-21, IL-23, IL-17E, IL-27; B cell immune mediators: BCA-1/CXCL13, SDF-1α+β/CXCL12, BAFF, APRIL.

In addition to assessing cytokine and chemokine concentrations in acute serum samples (S1) of VBT patients, levels of immune mediators were also measured in serum samples taken 2–3 weeks (S2) and 1–2 months (S3) after the onset of neurological symptoms. Of all measured immune mediators, only levels of IL-15 and MCP-1/CCL2 significantly differed between serum samples of VBT patients drawn at different time points with a tendency to reach levels of healthy controls over time (**Figure 4**). However, the differences in serum levels of proinflammatory (GROα/CXCL1, and IL-8), Th2 (IL-4), and B cell (BAFF, BCA-1/CXCL13, and SDF-1α+β/CXCL12) immune mediators between VBT TBE patients and healthy controls remained significant in S3 (up to two months after hospitalization) (p<0.05) (**Figure 4**).

These findings demonstrate a unique long lasting proinflammatory, Th2 and B cell immune response in patients with VBT TBE.

### Correlation Between Cytokine/Chemokine Concentrations and Leukocyte Count in Blood or CSF

As shown in **Table 4**, CSF concentrations of several innate and Th1 proinflammatory immune mediators (GM-CSF, IFN-α2, IL-10, IL-1RA, IL-1β, IL-6, IL-8, MIP-1α/CCL3, IFN-γ, IL-2) as well as Th2 cytokine (IL-5) and B cell-attracting chemokine 1 (BCA-1/CXCL13), tended to positively correlate with total leukocyte count in VBT patients. As for subpopulations of immune cells, innate and Th1 proinflammatory cytokines (GM-CSF, IFN-α2, IL-10, IL-1RA, IL-1β, IL-6, IL-8, and IL-2) as well as B cell-cytokine BAFF tended to positively correlate with CSF neutrophil counts, whereas other proinflammatory cytokines (IL-1β, TNF-α, MIP-3β/CCL9) as well as Th1 (FN-γ, MIG/CXCL9, IFN-λ3/IL-28β, IP-10/CXCL10, and I-TAC/CXCL11), Th2 (IL-5) and B-cell immune mediators (BCA-1/CXCL-13) tended to positively correlate with lymphocytes counts in CSF. BCA-1/CXCL-13 tended to positively correlate with monocyte counts in CSF, while IL-6 and IL-8 correlated negatively. However, these associations remained statistically significant after adjustment for multiple comparisons only in the case of IL-1β and total leukocyte count, IL-6 and IL-8 and neutrophil counts, and BCA-1/CXCL-13 and lymphocyte concentrations.

**Table 4:** Correlation of Cytokine/Chemokine levels in serum/CSF and leukocyte count in blood/CSF at hospitalization of VBT TBE patients.

Cytokines/Chemokines	Correlation with Leucocyte Count																							
	Blood												CSF											
	Leukocytes			Neutrophils			Lymphocytes			Monocytes			Leukocytes			Neutrophils			Lymphocytes			Monocytes		
	Rho^a^	p^b^	p Value Adjusted	Rho^a^	p^b^	p Value Adjusted	Rho^a^	p^b^	p Value Adjusted	Rho^a^	p^b^	p Value Adjusted	Rho^a^	p^b^	p Value Adjusted	Rho^a^	p^b^	p Value Adjusted	Rho^a^	p^b^	p Value Adjusted	Rho^a^	p^b^	p Value Adjusted
**Innate**																								
GM-CSF	0.032	0.847	1,000	0.027	0.872	1,000	0.039	0.818	1,000	0.149	0.378	1,000	0.367	**0.030**	0.422	0.366	**0.033**	0.412	0.125	0.482	1,000	-0.118	0.505	1,000
IFN-α2	-0.064	0.700	1,000	-0.078	0.648	1,000	0.084	0.622	1,000	0.029	0.864	1,000	0.356	**0.036**	0.477	0.374	**0.031**	0.387	0.243	0.167	0.965	0.05	0.779	1,000
GROα/CXCL1	0.093	0.578	1,000	0.187	0.267	1,000	-0.107	0.529	1,000	-0.248	0.139	0.986	0.323	0.059	0.658	0.308	0.076	0.728	0.262	0.134	0.924	-0.113	0.524	1,000
IL-10	0.214	0.196	0.998	0.266	0.112	0.966	-0.124	0.464	1,000	-0.179	0.289	1,000	0.452	**0.006**	0.113	0.393	**0.022**	0.299	0.052	0.771	1,000	-0.279	0.110	0.866
IL-15	0.081	0.629	1,000	0.153	0.366	1,000	-0.18	0.286	1,000	-0.080	0.639	1,000	0.066	0.708	1,000	0.244	0.164	0.95	-0.075	0.672	1,000	-0.255	0.145	0.939
IL-1RA	-0.085	0.612	1,000	-0.136	0.423	1,000	0.114	0.500	1,000	0.109	0.521	1,000	0.493	**0.003**	0.058	0.394	**0.021**	0.292	0.338	0.051	0.570	0.05	0.778	1,000
IL-1β	-0.296	0.071	0.867	-0.336	**0.042**	0.690	0.019	0.912	1,000	0.173	0.306	1,000	0.585	**<0.001**	**0.007**	0.377	**0.028**	0.369	0.376	**0.028**	0.384	<0.001	0.999	1,000
IL-6	0.093	0.579	1,000	0.102	0.549	1,000	-0.128	0.451	1,000	-0.064	0.705	1,000	0.398	**0.018**	0.270	0.536	**0.001**	**0.026**	-0.018	0.919	1,000	-0.354	**0.040**	0.471
IL-8	0.034	0.841	1,000	0.042	0.806	1,000	0.119	0.484	1,000	0.101	0.550	1,000	0.342	**0.044**	0.547	0.584	**<0.001**	**0.008**	-0.017	0.924	1,000	-0.415	**0.015**	0.220
MCP-1/CCL2	-0.142	0.397	1,000	-0.116	0.493	1,000	-0.039	0.820	1,000	0.059	0.729	1,000	0.012	0.943	1,000	0.236	0.179	0.962	-0.277	0.113	0.864	-0.306	0.079	0.743
MIP-1α/CCL3	-0.153	0.358	1,000	-0.18	0.288	1,000	0.185	0.274	1,000	0.201	0.232	1,000	0.462	**0.005**	0.097	0.303	0.082	0.751	0.136	0.442	1,000	-0.082	0.644	1,000
TNF-α	-0.146	0.380	1,000	-0.162	0.338	1,000	0.153	0.365	1,000	0.179	0.290	1,000	0.255	0.139	0.907	0.083	0.641	1,000	0.372	**0.030**	0.411	0.171	0.335	1,000
VEGF-A	0.280	0.089	0.916	0.32	0.053	0.783	0.141	0.406	1,000	-0.133	0.432	1,000	0.323	0.059	0.658	0.32	0.065	0.664	0.317	0.068	0.682	0.087	0.626	1,000
MIP-3β/CCL19	-0.006	0.969	1,000	0.019	0.911	1,000	0.033	0.845	1,000	-0.058	0.733	1,000	0.171	0.325	0.999	-0.069	0.700	1,000	0.44	**0.009**	0.154	0.176	0.320	1,000
IFN-β	-0.369	**0.023**	0.476	-0.329	**0.047**	0.736	-0.098	0.563	1,000	-0.032	0.852	1,000	-0.220	0.205	0.980	-0.023	0.896	1,000	-0.126	0.477	1,000	0.233	0.184	0.972
**Th1**																								
IFN-γ	-0.177	0.289	1,000	-0.281	0.092	0.937	0.212	0.208	1,000	0.402	**0.014**	0.338	0.411	**0.014**	0.212	0.306	0.078	0.733	0.34	**0.049**	0.558	0.06	0.735	1,000
IL-12 (p40)	-0.062	0.711	1,000	-0.065	0.702	1,000	-0.181	0.283	1,000	-0.085	0.616	1,000	0.204	0.240	0.986	-0.151	0.394	1,000	0.293	0.093	0.801	-0.224	0.202	0.991
IL-2	-0.083	0.620	1,000	-0.108	0.523	1,000	0.108	0.524	1,000	0.134	0.430	1,000	0.356	**0.036**	0.476	0.395	**0.021**	0.288	0.107	0.547	1,000	0.02	0.911	1,000
MIG/CXCL9	0.096	0.566	1,000	0.079	0.644	1,000	-0.065	0.701	1,000	0.097	0.567	1,000	0.069	0.692	1,000	-0.200	0.257	0.991	0.471	**0.005**	0.096	0.232	0.188	0.975
IFN-λ3/IL-28β	0.232	0.160	0.988	0.172	0.309	1,000	0.259	0.122	0.977	0.281	0.092	0.937	0.222	0.200	0.978	-0.011	0.951	1,000	0.396	**0.021**	0.306	0.114	0.522	1,000
IP-10/CXCL10	-0.071	0.672	1,000	-0.129	0.446	1,000	0.05	0.771	1,000	0.282	0.091	0.928	0.259	0.133	0.891	0.094	0.230	1,000	0.374	**0.030**	0.400	0.1	0.572	1,000
I-TAC/CXCL11	-0.063	0.708	1,000	-0.094	0.579	1,000	-0.026	0.88	1,000	0.196	0.244	1,000	0.174	0.318	0.999	-0.021	0.905	1,000	0.389	**0.023**	0.329	0.08	0.654	1,000
**Th2**																								
IL-4	-0.040	0.812	1,000	-0.019	0.911	1,000	0.116	0.495	1,000	0.050	0.767	1,000	0.325	0.057	0.649	0.211	0.460	0.988	-0.011	0.950	1,000	-0.237	0.177	0.972
IL-5	0.103	0.536	1,000	0.092	0.590	1,000	-0.076	0.657	1,000	-0.033	0.848	1,000	0.408	**0.015**	0.218	0.131	0.062	1,000	0.461	**0.006**	0.116	0.015	0.932	1,000
**Th17**																								
IL-17A	0.012	0.941	1,000	0.016	0.926	1,000	-0.032	0.852	1,000	0.119	0.483	1,000	0.324	0.058	0.654	0.323	0.647	0.647	0.177	0.317	0.999	-0.048	0.787	1,000
IL-17F	0.177	0.287	1,000	0.179	0.289	1,000	-0.186	0.272	1,000	-0.129	0.447	1,000	NA	NA	NA	NA	NA	NA	NA	NA	NA	NA	NA	NA
IL-22	0.116	0.487	1,000	0.083	0.627	1,000	-0.021	0.901	1,000	0.114	0.503	1,000	NA	NA	NA	NA	NA	NA	NA	NA	NA	NA	NA	NA
IL-21	0.058	0.732	1,000	0.097	0.567	1,000	-0.111	0.512	1,000	-0.063	0.712	1,000	-0.173	0.321	0.999	-0.116	0.515	1,000	-0.257	0.142	0.943	0.009	0.960	1,000
IL-23	0.284	0.084	0.909	0.230	0.171	0.997	0.102	0.547	1,000	0.132	0.435	1,000	NA	NA	NA	NA	NA	NA	NA	NA	NA	NA	NA	NA
IL-17E	0.210	0.206	0.999	0.203	0.228	1,000	-0.024	0.890	1,000	-0.008	0.963	1,000	NA	NA	NA	NA	NA	NA	NA	NA	NA	NA	NA	NA
IL-27	-0.036	0.831	1,000	0.024	0.888	1,000	-0.087	0.607	1,000	-0.115	0.498	1,000	0.024	0.891	1,000	-0.038	0.831	1,000	0.255	0.146	0.949	0.119	0.504	1,000
**B cell**																								
BCA-1/CXCL13	0.028	0.866	1,000	0.025	0.881	1,000	0.132	0.437	1,000	-0.032	0.852	1,000	0.342	**0.044**	0.548	-0.132	0.456	1,000	0.703	**<0.001**	**0.001**	0.42	**0.014**	0.212
SDF-1α+β/CXCL12	-0.130	0.436	1,000	-0.165	0.331	1,000	0.03	0.861	1,000	0.035	0.837	1,000	-0.136	0.436	1,000	-0.163	0.358	1,000	0.034	0.849	1,000	-0.037	0.835	1,000
BAFF	-0.308	0.060	0.816	-0.301	0.070	0.880	-0.057	0.739	1,000	0.120	0.481	1,000	0.256	0.137	0.902	0.374	**0.029**	0.381	0.254	0.147	0.950	0.092	0.605	1,000
APRIL	0.172	0.302	1,000	0.108	0.526	1,000	0.104	0.538	1,000	0.143	0.398	1,000	0.021	0.906	1,000	0.075	0.672	1,000	0.167	0.344	0.999	-0.021	0.906	1,000

^a^Spearman’s Rho-rank based correlation; values>0 indicate positive associations, values <0 indicate negative associations. ^b^The association was tested using Spearman’s method. Statistically significant p values (p<0.05) are in bold. CSF-cerebrospinal fluid. NA-not avaiable due to no variability in concentrations. CSF-cerebrospinal fluid.

On the contrary, associations between serum cytokine/chemokine concentrations and blood leukocyte counts were less pronounced and generally negatively correlated with cellular subsets. However, none of the association remained significant after adjustment for multiple comparisons.

Thus, whereas levels of innate and adaptive immune mediators generally correlated positively with white blood cell counts in CSF, these correlations tended to be negative in serum, implying that during early stages of neurological phase of TBE, cellular immune responses are likely mainly occurring locally within CNS.

### Correlation Between Cytokine/Chemokine Concentrations and Anti-TBEV IgG Antibody Levels in Serum or CSF

In CSF, the concentrations of most cytokines and chemokines tended to negatively correlate with anti-TBEV IgG antibodies, especially several innate mediators such as GM-CSF, βRA, IL-1β, IL-6, IL-8, and Th2 mediator IL-4. On the other hand, one mediator, B cell cytokine BAFF, tended to positively correlate with IgG levels in CSF of VBT patients. However, these associations remained statistically significant after adjustment for multiple comparisons only for IL-6 (**Table 5**).

**Table 5:** Correlation of Cytokine/Chemokine levels in serum/CSF and IgG antibodies in serum/CSF of VBT TBE patients.

Cytokines/Chemokines	Correlation with anti-TBEV IgG antobody levels												
		S1			S2			S3			CSF		
		Rho^a^	p^b^	p Value Adjusted	Rho^a^	p^b^	p Value Adjusted	Rho^a^	p^b^	p Value Adjusted	Rho^a^	p^b^	p Value Adjusted
**Innate**												
GM-CSF		-0.011	0.947	1.000	-0.224	0.385	1.000	0,253	0.257	1.000	-0.347	**0.044**	0.549
IFN-α2		-0.140	0.384	1.000	0.068	0.795	1.000	-0.158	0.482	1.000	-0.256	0.144	0.982
GROα/CXCL1		0.140	0.384	1.000	-0.127	0.625	1.000	0.293	0.186	0.998	-0.126	0.479	1.000
IL-10		-0.228	0.151	0.994	0.204	0.432	1.000	-0.049	0.830	1.000	-0.335	0.053	0.599
IL-15		0.097	0.548	1.000	-0.013	0.961	1.000	0.017	0.939	1.000	-0.269	0.125	0.899
IL-1RA		-0.295	0.061	0.815	-0.289	0.260	1.000	0.048	0.832	1.000	-0.402	**0.019**	0.287
IL-1β		-0.124	0.439	1.000	-0.361	0.155	0.984	-0.190	0.397	1.000	-0.469	**0.005**	0.090
IL-6		0.196	0.220	1.000	-0.041	0.877	1.000	0.185	0.411	1.000	-0.595	**<0.001**	**0.004**
IL-8		0.262	0.098	0.940	0.319	0.212	0.998	0.073	0.747	1.000	-0.480	**0.004**	0.068
MCP-1/CCL2		0.109	0.496	1.000	0.173	0.507	1.000	0.302	0.173	0.997	-0.226	0.199	0.974
MIP-1α/CCL3		0.042	0.792	1.000	0.217	0.403	1.000	-0.273	0.218	1.000	-0.261	0.136	0.912
TNF-α		0.073	0.650	1.000	0.373	0.140	0.973	0.227	0.310	1.000	-0.008	0.966	1.000
VEGF-A		-0.130	0.419	1.000	-0.079	0.764	1.000	0.211	0.347	1.000	-0.022	0.901	1.000
MIP-3β/CCL19		-0.201	0.207	1.000	-0.290	0.260	1.000	0.232	0.312	1.000	0.131	0.460	1.000
IFN-β		-0.105	0.513	1.000	0.112	0.669	1.000	0.215	0.337	1.000	0.224	0.203	0.980
**Th1**												
IFN-γ		0.058	0.720	1.000	-0.231	0.372	1.000	0.221	0.322	1.000	-0.251	0.153	0.939
IL-12 (p40)		0.091	0.571	1.000	0.000	1.000	1.000	-0.053	0.814	1.000	-0.275	0.115	0.877
IL-2		-0.144	0.369	1.000	-0.216	0.406	1.000	0.315	0.153	0.997	-0.247	0.159	0.946
MIG/CXCL9		0.280	0.076	0.879	0.534	**0.029**	0.512	0.126	0.586	1.000	0.300	0.084	0.772
IFN-λ3/IL-28β		0.147	0.359	1.000	NA	NA	NA	-0.237	0.289	1.000	-0.026	0.885	1.000
IP-10/CXCL10		0.166	0.299	1.000	0.168	0.519	1.000	-0.013	0.954	1.000	-0.133	0.453	1.000
I-TAC/CXCL11		-0.129	0.420	1.000	-0.248	0.337	1.000	-0.324	0.141	0.993	0.119	0.501	1.000
**Th2**												
IL-4		0.002	0.990	1.000	0.175	0.501	1.000	0.585	**0.004**	0.104	-0.462	**0.006**	0.108
IL-5		0.123	0.443	1.000	0.059	0.821	1.000	0.210	0.348	1.000	-0.068	0.702	1.000
**Th17**												
IL-17A		0.297	0.059	0.810	0.263	0.309	1.000	0.393	0.070	0.871	-0.294	0.092	0.799
IL-17F		-0.029	0.859	1.000	-0.153	0.557	1.000	-0.376	0.085	0.907	NA	NA	NA
IL-22		0.115	0.474	1.000	-0.051	0.846	1.000	-0.376	0.370	1.000	NA	NA	NA
IL-21		0.214	0.180	0.999	-0.428	0.086	0.879	-0.039	0.865	1.000	-0.009	0.960	1.000
IL-23		-0.017	0.918	1.000	0.010	0.970	1.000	-0.067	0.769	1.000	NA	NA	NA
IL-17E		-0.241	0.130	0.985	NA	NA	NA	-0.292	0.187	0.998	NA	NA	NA
IL-27		0.211	0.185	1.000	-0.446	0.072	0.829	0.344	0.117	0.968	-0.079	0.657	1.000
**B cell**												
BCA-1/CXCL13		-0.156	0.331	1.000	-0.301	0.240	1.000	-0.106	0.637	1.000	0.208	0.238	0.991
SDF-1α+β/CXCL12		-0.159	0.321	1.000	-0.647	**0.005**	0.117	-0.159	0.479	1.000	-0.019	0.913	1.000
BAFF		-0.011	0.944	1.000	-0.136	0.602	1.000	0.023	0.918	1.000	0.348	**0.044**	0.544
APRIL		-0.020	0.899	1.000	-0.513	**0.035**	0.578	0.283	0.202	0.999	-0.054	0.763	1.000

^a^Spearman’s Rho-rank based correlation; values>0 indicate positive associations, values <0 indicate negative associations. ^b^The association was tested using Spearman’s method. Statistically significant p values (p<0.05) are in bold. NA-not avaiable due to no variability in concentrations. CSF-cerebrospinal fluid. S-serum.

S1 and CSF values were determined in serum and CSF samples taken at hospitalization, S2 in serum samples taken from two to three weeks and S3 in serum samples taken one to two month after hospitalization.

In contrast to CSF, no significant correlations were observed between immune mediators and IgG antibody levels in serum samples obtained on admission. In sera obtained two to three weeks after hospitalization, only MIG/CXCL9 levels correlated positively with serum IgG antibodies, whereas SDF-1α+β/CXCL12 and APRIL correlated negatively. At one to two months after hospital discharge, only IL-4 correlated positively with IgG antibody levels (**Table 5**), but the associations did not remain significant after adjustment for multiple comparisons.

The observed negative correlation between innate inflammatory mediators and anti-TBEV IgG antibodies in CSF, at the onset of neurological symptoms in VBT patients, indicate the dominance of inflammatory immune responses in CNS without the corresponding increase in humoral responses to TBEV infection. This immune imbalance may contribute to TBE neuropathology.

## Discussion

Although a robust immune response is crucial for early virus clearance (King et al., 2007; Lindqvist et al., 2016), many studies have proposed its direct involvement in pathological manifestations of TBEV infection (Lepej et al., 2007; Gunther et al., 2011; Kang et al., 2013; Grygorczuk et al., 2017; Grygorczuk et al., 2018; Bogovic et al., 2019). As vaccine breakthrough patients more often develop a monophasic disease with severe clinical manifestations and are hospitalized for a longer period in comparison to age and gender matched unvaccinated TBE patients (Lotric-Furlan et al., 2017), we aimed to investigate the role of immune mediators in pathogenesis of TBE in patients previously vaccinated against this disease. Since the first phase of the disease is characterized by non-specific symptoms and patients rarely seek medical help, we were only able to study cytokine and chemokine responses after TBEV entry to CNS during the 2nd phase when the neurological symptoms appeared. Nonetheless, the design of the study enabled an in-depth analysis of cytokines and chemokines reflecting inflammatory and adaptive immune responses early after neuroinvasion. Assessment of inflammatory mediators in serum and CSF on admission and in the convalescent phase of the disease allowed insights into innate and adaptive cellular immunity during TBE in patients previously vaccinated against this disease. VBT patients had generally higher concentrations of innate and Th1 adaptive mediators in CSF compared to serum (**Table 3** and **Figure 2**), which correlated positively with leukocyte counts (**Table 4**), suggesting that these responses are occurring locally within the CNS, the site of disease. Additionally, an observed negative correlation between innate inflammatory mediators and anti-TBEV IgG antibodies in CSF (**Table 5**) indicate the formation of predominantly inflammatory instead of humoral immune response, possibly contributing to TBE neuropathology.

The hallmark of TBE is the breakdown of BBB that corresponds with overexpression of proinflammatory mediators in the brain as well as with disease progression (Ruzek et al., 2011). One of the factors that can alter BBB integrity is VEGF, a known stimulator of vasculoneogenesis and angiogenesis. VEGF overexpression increases the metalloproteinase (MMP)-9 activity, leading to degradation of extracellular matrix proteins and increased permeability of CNS vasculature (Valable et al., 2005). Elevated expression of VEGF and MMP-9 was previously associated with CNS inflammatory reactions, BBB disruption and disease severity in TBE patients (Kang et al., 2013; Palus et al., 2014; Palus et al., 2015). We postulate that the prominent systemic and intrathecal upregulation of VEGF-A observed in VBT patients in our study, suggests an extensive BBB impairment that facilitates the entry of immune cells into CNS and the formation of intrathecal inflammatory focus. This observation offers a potential explanation for the more severe clinical manifestation of the disease reported for VBT patients (Lotric-Furlan et al., 2017).

Along with VEGF-A, proinflammatory cytokines were also shown to alter BBB integrity in different brain disorders, including TBE (Almutairi et al., 2016; Mustafa et al., 2019) and were also strongly upregulated and concentrated in CSF of TBE patients at the onset of neurological symptoms (Grygorczuk et al., 2006; Zajkowska et al., 2011; Grygorczuk et al., 2017; Grygorczuk et al., 2018; Koper et al., 2018; Bogovic et al., 2019). Similarly as previously reported for nonvaccinated TBE patients, we observed a systemic upregulation of some proinflammatory cytokines in VBT patients, yet the majority of them, including IFN-α2, IFN-β, IL-6, IL-8, IL-15, MCP-1/CCL-2, and MIP-3β/CCL19, was significantly higher in CSF, suggesting their upregulated production at the primary site of infection in the 2nd phase of the disease. Three of the proinflammatory cytokines, IL-6, IL-8 and MCP-1/CCL-2, are potentially capable of disrupting BBB permeability through alteration of tight-junction protein expression or promotion of MMP-9 activity (Kang et al., 2013) facilitating immune cell trafficking into the CNS and promoting CNS inflammation. IL-8 is also known to attract T cells and neutrophils to the site of infection. With the ability of TBEV to infect neutrophils (Plekhova et al., 2012) and a tendency toward positive association of IL-8 levels with total pleocytosis and CSF neutrophil counts observed in VBT patients, IL-8 expression could significantly contribute to virus dissemination and CNS immunopathology during TBE in patients previously vaccinated against this disease. Also, an observed higher expression of IL-8 and GRO-α/CXCL1 in convalescent sera of VBT patients compared to serum levels of healthy controls, point to a lengthened disruption of BBB, preventing CSF hemostasis and disease remission. Additionally, IFN-γ together with IFN-γ inducible chemoattractants, MIG/CXCL9, and IP-10/CXCL10 as well as MIP-3β/CCL19, a T and dendritic cell chemoattractants, were also upregulated intrathecally in VBT patients and tended to positively correlate with immune cell count in CSF, especially with lymphocytes, but did not correlate with leukocytes in sera, proposing their important role in immune cell trafficking in CNS. A positive correlation between MIG/CXCL9, IP-10/CXCL10, I-TAC/CXCL11 and MIP-3β/CCL19 and mononuclear cell infiltrates was also observed in previous studies on TBE patients, confirming their potential involvement in lymphocyte extravasation to the site of infection during TBE (Bogovic et al., 2019).

Besides upregulated intrathecal expression of inflammatory innate and Th1 cytokines detected in VBT TBE patients at the onset of neurological symptoms, the observed negative correlation between these immune mediators and anti-TBEV IgG antibodies in CSF additionally supports the hypothesis of a disproportionally stronger and possibly harmful inflammatory immune response, relative to antibody mediated protective response in CNS. Interestingly, higher intrathecal expression of IFN-β was observed in patients with milder clinical presentation (Grygorczuk et al., 2015), yet our results showed a significantly higher CSF level of IFN-β in VBT patients, known to develop a more severe disease, compared to unvaccinated TBE patients. This finding indicates a more robust primary immune response to a still active viral infection or a countermeasure to augmented CNS injury in VBT patients with a more severe disease manifestation.

In addition to evident upregulation of proinflammatory cytokines observed in VBT patients at the beginning of neurological phase and later on, the results of our study also suggest an involvement of Th2 and B cell responses in pathogenesis of TBE in patients previously vaccinated against this disease. We observed a distinct distribution of Th2 and B cell cytokines in VBT patients, as IL-4 and BAFF levels were concentrated in serum and were significantly higher than in healthy controls, whilst IL-5 and APRIL were concentrated in CSF. These results indicate that upregulated antibody production through stimulation of plasma cells, involving these immune mediators (Vincent et al., 2013; Moens and Tangye, 2014), occurs in both compartments, leading to extensive humoral response in VBT patients at admission (Stiasny et al., 2009; Lotric-Furlan et al., 2017). Surprisingly, even with significantly higher humoral response observed in VBTs previously (Stiasny et al., 2009; Lotric-Furlan et al., 2017), we found no differences in APRIL and BAFF concentrations between vaccinated and unvaccinated TBE patients. One possible explanation could be a major upregulation of APRIL and BAFF expression earlier in the disease progression supporting the formation of already established antibody response observed at the onset of neurological symptoms in VBT patients. Also, serum levels of Th2 and B cell cytokines IL-4 and BAFF, remained elevated up to two months after the onset of neurological symptoms in VBT patients, presumably supporting the extensive IgG and a delayed IgM antibody production observed in majority of VBT patients (Stiasny et al., 2009; Lotric-Furlan et al., 2017). Occurrence of IgM production despite previous encounter with TBEV antigens *via* vaccination could be due to inaccessibility of some TBEV epitopes in inactivated vaccine formulations that challenge the immune system for the first time in the course of natural infection with TBEV.

In conclusion, despite highly immunogenic and efficient vaccine against TBE, some vaccine breakthroughs have been reported in several European countries. Although a few studies characterizing humoral response in VBT patients exist, there is limited information available on other immunological features. Our results show that the onset of neurological symptoms in VBT patients is associated with global elevation of innate immune mediators, especially those associated with neutrophil activation and chemotaxis. In addition to CSF levels of other innate and Th1 immune mediators, the CSF levels of IFN-β and VEGF-A -immune mediators that promote BBB breakdown and, in this way, facilitate immune cell influx into the CNS- are especially pronounced. Additionally, a systemic upregulation of IL-8 and GROα/CXCL1 expression is present even two months after the onset of neurological symptoms in VBT patients, suggesting a sustained neutrophil driven inflammatory response with a prolonged disruption of BBB, which could contribute to disease progression and remission while preventing CSF hemostasis. On the other hand, cytokines and chemokines reflecting Th2 and B cell responses are upregulated in both, serum and CSF of VBT patients at the beginning of neurological phase, indicating the formation of humoral response in both compartments. Additionally, a prolonged upregulation of IL-4 and BAFF and a positive correlation of IL-4 with IgG antibodies measured two months later, suggests a long lasting humoral immune response, possibly connected to a delayed IgM antibody response, a phenomenon previously observed in VBT TBE patients.

These findings provide a first insight into immunopathogenesis of TBE in patients previously vaccinated against this disease. An observed prominent systemic and intrathecal upregulation of VEGF-A in VBT patients suggests extensive BBB impairment that facilitates the entry of immune cells into CNS and the formation of intrathecal inflammatory focus resulting in more severe clinical manifestation as previously reported for VBT patients. Additional studies on specific cell subsets and their involvement in TBE pathogenesis in this group of patients are needed to further elucidate the mechanism behind the phenomenon of vaccine breakthrough infection.

## Data Availability Statement

The raw data supporting the conclusions of this article will be made available by the authors, without undue reservation.

## Ethics Statement

The studies involving human participants were reviewed and approved by National Medical Ethic Committee (approval number 131/06/13, 0120-188/2018/6, 81/12/2013, 0120-188/2018/6, and 0120-564/2018/13). The patients/participants provided their written informed consent to participate in this study.

## Author Contributions

MP, MK, NK, and TAZ designed the study. MP and MK performed the experiments and formal analysis. LL, MP, and MK did the data analysis and interpretation. MP wrote the original draft. MP, MK, NK, TZ, PB, KS, FS, LL, MNK, TV, JT, and SL-F performed review and editing. SL-F, MNK, TV, JT, PB, and FS helped in collecting of the clinical samples. All authors contributed to the article and approved the submitted version.

## Funding

This research was funded by the Slovenian Research Agency, grant numbers P3-0083 and P3-0296.

## Conflict of Interest

PB served on the scientific advisory board for Pfizer on TBE CEE expert meeting 2020. KS served on the scientific advisory board for Roche for development of Lyme disease serological diagnostics received support by grant from MGH-ECOR. FS served on the scientific advisory board for Roche on Lyme disease serological diagnostics and the scientific advisory board for Pfizer on TBE CEE expert meeting 2020, has been a member of Pfizer Lyme Vaccine Licensure Advisory Board, received research support from the Slovenian Research Agency [grant number P3-0296], and is an unpaid member of the steering committee of the ESCMID Study Group on Lyme Borreliosis/ESGBOR.

The remaining authors declare that the research was conducted in the absence of any commercial or financial relationships that could be construed as a potential conflict of interest.

## Footnotes

1 https://www.nijz.si/sl/pravocasno-se-zascitimo-pred-klopi-saj-lahko-prenasajo-bolezni-3

## Abbreviations

APRIL, a proliferation-inducing ligand; BAFF, B-cell activating factor; BBB, blood-brain barrier; BCA, B cell-attracting chemokine; CNS, central nervous system; CSF, cerebrospinal fluid; GM-CSF, granulocyte macrophage colony stimulating factor; I-TAC, interferon-inducible T-cell alpha chemoattractant; IFN, interferon; Ig, immunoglobulin; IL, interleukin; IP-10, inducible protein 10; MCP, macrophage chemotactic protein 1; MIG, monokine induced by gamma interferon; MIP, macrophage inflammatory protein; SDF, stromal cell-derived factor; TNF, tumor necrosis factor; Th, T helper cell; TBE, tick-borne encephalitis; TBEV, tick-borne encephalitis virus; VBT, vaccine breakthrough; VEGF, vascular endothelial growth factor.
