# Supplementary material for: Upregulated Intrathecal Expression of VEGF-A and Long Lasting Global Upregulation of Proinflammatory Immune Mediators in Vaccine Breakthrough Tick-Borne Encephalitis
Source: Front Cell Infect Microbiol. 2021 Jul 1;11:696337. doi: 10.3389/fcimb.2021.696337 (PMC8281926; doi:10.3389/fcimb.2021.696337)
Supplement: Supplementary file 1 [file DataSheet_1.docx]

Supplementary Material

**Supplementary table 1.** Concentrations of cytokines/chemokines in serum and CSF samples of VBT patients with incomplete and complete vaccination against TBE. Significantly higher concentrations of cytokines in each group and the corresponding p values are shown in bold.

|  | **Concentrations (pg/ml) Median (IQR)** | | | | | | | |
| --- | --- | --- | --- | --- | --- | --- | --- | --- |
|  | **serum** | | | | **CSF** | | | |
|  | **incomplete** | **complete** | **p Value** | **p Value Adjusted** | **incomplete** | **complete** | **p Value** | **p Value Adjusted** |
| **Innate** |  |  |  |  |  |  |  |  |
| GM-CSF | 0.79 (0.33-1.66) | 1.21 (0.79-2.30) | 0.083 | 0.865 | 1.54 (0.79-3.90) | 1.28 (0.79-1.79) | 0.615 | 1.000 |
| IFN-α2 | 11.05 (4.63-21.04) | 11.05 (4.63-24.95) | 0.423 | 1.000 | 43.35 (32.30-54.37) | 39.08 (27.50-45.45) | 0.212 | 0.992 |
| GROα/CXCL1 | 512.86 (233.96-830.45) | 440.98 (312.14-1020.88) | 0.899 | 1.000 | 0.30 (0.30-3.24) | 0.30 (0.30-23.20) | 0.325 | 0.997 |
| IL-10 | 0.12 (0.12-0.35) | 0.12 (0.12-0.16) | 1.000 | 1.000 | 0.12 (0.12-0.38) | 0.12 (0.12-0.12) | 0.551 | 1.000 |
| IL-15 | 0.13 (0.13-0.32) | 0.42 (0.32-1.06) | **0.045** | 1.000 | 0.86 (0.49-2.62) | 2.42 (0.93-4.10) | 0.430 | 1.000 |
| IL-1RA | 0.40 (0.16-1.86) | 0.16 (0.16-5.16) | 0.600 | 0.674 | 1.22 (0.16-9.22) | 0.16 (0.16-4.16) | 0.598 | 1.000 |
| IL-1β | 0.37 (0.37-0.65) | 0.37 (0.37-0.37) | 0.127 | 1.000 | 0.370 (0.370-0.370) | 0.37 (0.37-0.37) | 0.314 | 0.997 |
| IL-6 | 0.44 (0.23-2.16) | 0.23 (0.23-0.23) | 0.136 | 0.961 | 79.12 (3.39-777.44) | 112.64 (25.39-261.99) | 0.942 | 1.000 |
| IL-8 | 6.69 (1.79-165.31) | 2.96 (0.83-9.91) | 0.182 | 0.991 | 41.35 (17.61-96.15) | 42.69 (14.00-79.59) | 0.760 | 1.000 |
| MCP-1/CCL2 | 84.95 (66.87-139.60) | 88.44 (64.54-137.79) | 0.989 | 1.000 | 167.81 (83.66-564.28) | 180.01 (94.25-432.31) | 0.760 | 1.000 |
| MIP-1α/CCL3 | 7.03 (0.36-77.41) | 0.36 (0.36-7.51) | 0.093 | 1.000 | 0.36 (0.36-0.36) | 0.36 (0.36-0.36) | **0.044** | 0.527 |
| TNF-α | 12.17 (6.52-34.49) | 7.14 (4.35-11.55) | **0.042** | 0.896 | 3.39 (2.16-3.87) | 3.39 (2.16-4.83) | 0.826 | 1.000 |
| VEGF-A | 66.32 (43.16-80.13) | 58.18 (52.24-88.73) | 0.897 | 0.646 | 40.44 (29.16-44.78) | 38.58 (25.63-45.78) | 0.348 | 1.000 |
| MIP-3β/CCL19 | 9.89 (9.89-14.82) | 9.89 (9.89-14.82) | 0.654 | 1.000 | 97.69 (24.95-123.72) | 134.03 (89.70-194.80) | 0.201 | 0.990 |
| INF-β | 33.84 (33.84-33.84) | 33.84 (33.84-33.84) | 0.705 | 1.000 | 47.33 (33.84-103.04) | 33.84 (33.84-103.04) | 0.422 | 1.000 |
| **Th1** |  |  |  |  |  |  |  |  |
| IFN-γ | 1.05 (0.62-2.95) | 1.31 (0.62-2.69) | 0.989 | 1.000 | 21.75 (6.57-38.86) | 16.72 (8.55-30.72) | 0.956 | 1.000 |
| IL-12(p40) | 0.15 (0.15-0.15) | 0.15 (0.15-0.15) | 0.377 | 1.000 | 0.15 (0.15-0.15) | 0.15 (0.15-0.15) | 0.569 | 1.000 |
| IL-2 | 0.33 (0.33-0.49) | 0.33 (0.33-0.44) | 0.812 | 1.000 | 0.53 (0.33-0.96) | 0.33 (0.33-0.53) | 0.360 | 1.000 |
| MIG/CXCL9 | 127.70 (75.50-186.57) | 137.62 (82.10-20.90) | 0.731 | 1.000 | 317.33 (164.68-488.21) | 351.63 (284.18-661.77) | 0.627 | 1.000 |
| INF-λ3/IL-28β | 2.11 (2.11-2.11) | 2.11 (2.11-2.11) | 0.555 | 1.000 | 2.11 (2.11-2.11) | 2.11 (2.11-2.11) | 0.508 | 1.000 |
| IP-10/CXCL10 | 100.81 (80.79-148.46) | 127.53 (62.78-179.17) | 0.989 | 1.000 | 7505.50 (2928.00-14193.25) | 8056 (5099-15586) | 0.760 | 1.000 |
| I-TAC/CXCL11 | 24.19 (12.76-34.13) | 25.20 (12.76-34.13) | 0.557 | 1.000 | 7.69 (1.99-17.70) | 12.77 (6.72-22.11) | 0.520 | 1.000 |
| **Th2** |  |  |  |  |  |  |  |  |
| IL-4 | 28.32 (12.38-85.16) | 116.90 (33.18-321.04) | 0.049 | 0.720 | 0.31 (0.31-2.19) | 0.31 (0.31-0.31) | 0.338 | 0.999 |
| IL-5 | 0.20 (0.20-0.20) | 0.20 (0.20-0.20) | 0.899 | 1.000 | 0.47 (0.20-1.02) | 0.20 (0.20-1.39) | 0.332 | 0.997 |
| **Th17** |  |  |  |  |  |  |  |  |
| IL-17A | 0.82 (0.43-1.58) | 0.58 (0.35-0.99) | 0.200 | 1.000 | 0.76 (0.47-1.11) | 0.70 (0.58-0.81) | 0.402 | 1.000 |
| IL-17F | 10 (10-10) | 10 (10-10) | 0.539 | 1.000 | 10 (10-10) | 10 (10-10) | NA | NA |
| IL-22 | 20 (20-20) | 20 (20-20) | 0.889 | 1.000 | 20 (20-20) | 20 (20-20) | NA | NA |
| IL-21 | 2.72 (2.72-4.55) | 2.72 (2.72-7.55) | 0.625 | 1.000 | 2.72 (2.27-2.27) | 2.72 (2.27-2.27) | 0.389 | 1.000 |
| IL-23 | 80 (80-80) | 80 (80-80) | 0.836 | 1.000 | 80 (80-80) | 80 (80-80) | NA | NA |
| IL-17E | 60 (60-60) | 60 (60-60) | 0.133 | 0.991 | 60 (60-60) | 60 (60-60) | NA | NA |
| IL-27 | 180 (110-290) | 180 (140-270) | 0.954 | 1.000 | 100 (30-190) | 120 (80-250) | 0.420 | 1.000 |
| **B cell** |  |  |  |  |  |  |  |  |
| BCA-1/CXCL13 | 5.8 (3.26-8.04) | 5.18 (1.33-7.23) | 0.508 | 1.000 | 1.66 (0.27-9.03) | 1.78 (0.27-13.47) | 0.932 | 1.000 |
| SDF-1α+β/CXCL12 | 585.080 (520.86-629.72) | 602.20 (530.27-711.61) | 0.251 | 1.000 | 464.91 (200.08-698.58) | 768.69 (501.58-921.43) | 0.086 | 0.727 |
| BAFF | 0.30 (0.21-0.47) | 0.31 (0.16-0.43) | 0.697 | 1.000 | 0.06 (0.06-0.11) | 0.06 (0.06-0.11) | 0.984 | 1.000 |
| APRIL | 254.73 (155.98-368.44) | 254.73 (206.41-346.39) | 0.384 | 1.000 | 495.09 (242.65-671.83) | 671.83 (505.30-863.89) | 0.159 | 0.950 |

CSF-cerebrospinal fluid. NA-not avaiable due to no variability in concentrations.

**Supplementary table 2.** Concentrations of cytokines/chemokines in serum and CSF samples of VBT patients according to gender. Significantly higher concentrations of cytokines in each group and the corresponding p values are shown in bold.

|  | **Concentration (pg/ml) Median (IQR)** | | | | | | | |
| --- | --- | --- | --- | --- | --- | --- | --- | --- |
|  | **serum** | | | | **CSF** | | | |
|  | **male** | **female** | **p** | **p Value Adjusted** | **male** | **female** | **p** | **p Value Adjusted** |
| **Innate** |  |  |  |  |  |  |  |  |
| GM-CSF | 0.79 (0.45-1.66) | 1.79 (1.04-3.10) | **0.013** | 0.250 | 1.79 (0.79-2.31) | 1.28 (0.79-1.92) | 0.535 | 1.000 |
| IFN-α2 | 11.05 (4.63-16.95) | 16.95 (4.63-24.95) | 0.188 | 0.996 | 41.25 (29.90-51.46) | 34.59 (29.83-45.96) | 0.398 | 1.000 |
| GROα/CXCL1 | 431.36 (287.52-726.68) | 459.00 (305.58-1416.50) | 0.325 | 1.000 | 0.30 (0.30-0.30) | 0.30 (0.30-58.17) | 0.162 | 1.000 |
| IL-10 | 0.12 (0.12-0.17) | 0.12 (0.12-0.28) | 0.874 | 1.000 | 0.12 (0.12-0.16) | 0.12 (0.12-0.14) | 0.949 | 1.000 |
| IL-15 | 0.32 (0.32-0.67) | 0.32 (0.32-0.80) | 0.965 | 1.000 | 2.42 (0.74-4.27) | 0.99 (0.49-3.26) | 0.335 | 1.000 |
| IL-1RA | 0.16 (0.16-2.27) | 0.64 (0.16-5.16) | 0.325 | 1.000 | 0.16 (0.16-10.25) | 0.16 (0.16-5.30) | 0.626 | 1.000 |
| IL-1β | 0.37 (0.37-0.37) | 0.37 (0.37-0.57) | 0.241 | 1.000 | 0.37 (0.37-0.62) | 0.37 (0.37-0.50) | 0.693 | 1.000 |
| IL-6 | 0.23 (23-0.78) | 0.23 (0.23-1.39) | 0.608 | 1.000 | 96.84 (25.25-327.24) | 87.02 (3.16-274.38) | 0.708 | 1.000 |
| IL-8 | 2.96 (1.03-9.95) | 3.18 (0.84-31.46) | 0.781 | 1.000 | 45.20 (23.21-81.49) | 22.14 (10.49-110.49) | 0.538 | 1.000 |
| MCP-1/CCL2 | 78.78 (64.12-145.55) | 88.44 (66.67-137.79) | 0.624 | 1.000 | 180.01 (92.26-372.77) | 176.25 (92.09-451.63) | 0.803 | 1.000 |
| MIP-1α/CCL3 | 0.36 (0.36-7.76) | 0.36 (0.36-28.94) | 0.625 | 1.000 | 0.36 (0.36-0.36) | 0.36 (0.36-0.36) | 0.696 | 1.000 |
| TNF-α | 8.27 (4.94-13.25) | 6.46 (4.77-20.12) | 0.968 | 1.000 | 3.39 (2.41-4.83) | 3.15 (2.10-5.58) | 0.745 | 1.000 |
| VEGF-A | 59.58 (43.16-78.43) | 66.32 (52.24-98.25) | 0.320 | 1.000 | 38.58 (27.98-45.78) | 32.48 (25.63-45.78) | 0.486 | 1.000 |
| MIP-3β/CCL19 | 9.89 (9.89-19.43) | 9.89 (9.89-12.72) | 0.334 | 1.000 | 169.12 (64.02-252.78) | 97.69 (36.29-143.55) | 0.177 | 1.000 |
| INF-β | 33.84 (33.84-33.84) | 33.84 (33.84-33.84) | 0.944 | 1.000 | 33.84 (33.84-103.04) | 47.33 (33.84-103.04) | 0.694 | 1.000 |
| **Th1** |  |  |  |  |  |  |  |  |
| IFN-γ | 0.96 (0.62-2.61) | 2,00 (0.46-3.22) | 0.576 | 1.000 | 23.48 (9.58-41.30) | 10.77 (3.22-47.61) | 0.289 | 1.000 |
| IL-12(p40) | 0.15 (0.15-0.15) | 0.15 (0.15-0.15) | 0.859 | 1.000 | 0.15 (0.15-0.15) | 0.15 (0.15-0.15) | 0.400 | 1.000 |
| IL-2 | 0.33 (0.33-0.49) | 0.33 (0.33-0.44) | 0.481 | 1.000 | 0.53 (0.33-1.01) | 0.34 (0.33-0.58) | 0.589 | 1.000 |
| MIG/CXCL9 | 134.66 (83.32-206.55) | 116.47 80.92-202.00) | 0.691 | 1.000 | 351.63 (231.37-757.56) | 347.85 (259.84-649.60) | 1.000 | 1.000 |
| INF-λ3/IL-28β | 2.11 (2.11-2.11) | 2.11 (2.11-2.11) | 0.255 | 1.000 | 2.11 (2.11-2.11) | 2.11 (2.11-2.11) | 1.000 | 1.000 |
| IP-10/CXCL10 | 118.31 (63.11-171.02) | 104.58 (78.66-162.45) | 0.843 | 1.000 | 10109 (5086.50-17116.50) | 5790.50 (3558.25-15391) | 0.454 | 1.000 |
| I-TAC/CXCL11 | 19.89 (19.,01-41.31) | 19.89 (11.21-34.13) | 0.223 | 0.999 | 12.77 (6.72-24.62) | 6.72 (1.99-23.13) | 0.407 | 1.000 |
| **Th2** |  |  |  |  |  |  |  | 1.000 |
| IL-4 | 88.31 (17.2-166.33) | 71.34 (13.95-326.01) | 0.761 | 1.000 | 0.31 (0.31-0.31) | 0.31 (0.31-0.31) | 0.696 | 1.000 |
| IL-5 | 0.20 (0.20-0.20) | 0.20 (0.20-0.20) | 0.379 | 1.000 | 0.25 (0.20-1.69) | 0.23 (0.20-1.81) | 0.894 | 1.000 |
| **Th17** |  |  |  |  |  |  |  | 1.000 |
| IL-17A | 0.58 (0.38-1.04) | 0.58 (0.35-1.34) | 0.863 | 1.000 | 0.70 (0.58-0.99) | 0.70 (0.47-0.84) | 0.514 | 1.000 |
| IL-17F | 10 (10-10) | 10 (10-10) | 0.832 | 1.000 | 10 (10-10) | 10 (10-10) | 1.000 | 1.000 |
| IL-22 | 20 (20-20) | 20 (20-20) | 0.817 | 1.000 | 20 (20-20) | 20 (20-20) | 1.000 | 1.000 |
| IL-21 | 2.72 (2.72-4.04) | 3.18 (2.72-9.14) | 0.121 | 0.961 | 2.72 (2.27-2.27) | 2.72 (2.72-2.72) | 0.153 | 1.000 |
| IL-23 | 80 (80-80) | 80 (80-80) | 0.541 | 1.000 | 80 (80-80) | 80 (80-80) | 1.000 | 1.000 |
| IL-17E | 60 (60-60) | 60 (60-60) | 0.428 | 1.000 | 60 (60-60) | 60 (60-60) | 1.000 | 1.000 |
| IL-27 | 160 (110-240) | 260 (170-280) | **0.041** | 0.632 | 130 (40-240) | 110 (70-280) | 0.671 | 1.000 |
| **B cell** |  |  |  |  |  |  |  | 1.000 |
| BCA-1/CXCL13 | 3.91 (1.10-7.31) | 5.69 (4.26-7.23) | 0.433 | 1.000 | 2.49 (0.27-12.35) | 0.27 (0.27-22.59) | 0.965 | 1.000 |
| SDF-1α+β/CXCL12 | 585.08 (528.76-682.91) | 629.72 (538.95-698.64) | 0.741 | 1.000 | 768.69 (433.79-906.59) | 599.74 (337.39-936.36) | 0.961 | 1.000 |
| BAFF | 0.32 (0.18-0.50) | 0.24 (0.16-0.35) | 0.247 | 1.000 | 0.09 (0.06-0.12) | 0.06 (0.06-0.11) | 0.357 | 1.000 |
| APRIL | 254.73 (206.41-368.44) | 254.73 (18.67-346.39) | 0.799 | 1.000 | 594.97 (400.89-889.85) | 652.77 (474.65-863.89) | 0.940 | 1.000 |

CSF-cerebrospinal fluid.

**Supplementary table 3.** Correlation of Cytokine/Chemokine levels in serum of VBT patients at hospitalization with age. Significantly higher concentrations of cytokines in each group and the corresponding p values are shown in bold.

|  | **Correlation of Cytokine/Chemokine concentrations with age** | | | | | |
| --- | --- | --- | --- | --- | --- | --- |
|  | **serum** | | | **CSF** | | |
|  | **Rho** | **p** | **p Value Adjusted** | **Rho** | **p** | **p Value Adjusted** |
| **Innate** |  |  |  |  |  |  |
| GM-CSF | -0.011 | 0.850 | 1.000 | -0.019 | 0.913 | 1.000 |
| IFN-α2 | -0.005 | 0.939 | 1.000 | 0.007 | 0.970 | 1.000 |
| GROα/CXCL1 | 0.073 | 0.338 | 1.000 | -0.092 | 0.601 | 1.000 |
| IL-10 | 0.309 | 0.296 | 1.000 | -0.095 | 0.587 | 1.000 |
| IL-15 | 0.407 | 0.118 | 0.954 | 0.331 | 0.052 | 1.000 |
| IL-1RA | -0.008 | 0.960 | 1.000 | -0.125 | 0.475 | 1.000 |
| IL-1β | -0.048 | 0.534 | 1.000 | -0.148 | 0.396 | 1.000 |
| IL-6 | 0.059 | 0.923 | 1.000 | 0.202 | 0.244 | 1.000 |
| IL-8 | 0.262 | 0.695 | 1.000 | 0.232 | 0.180 | 1.000 |
| MCP-1/CCL2 | -0.142 | 0.456 | 1.000 | 0.245 | 0.156 | 1.000 |
| MIP-1α/CCL3 | 0.122 | 0.433 | 1.000 | -0.252 | 0.144 | 1.000 |
| TNF-α | 0.043 | 0.746 | 1.000 | -0.285 | 0.097 | 1.000 |
| VEGF-A | 0.144 | 0.244 | 1.000 | -0.183 | 0.294 | 1.000 |
| MIP-3β/CCL19 | -0.341 | 0.345 | 1.000 | -0.316 | 0.065 | 1.000 |
| INF-β | -0.172 | 0.448 | 1.000 | 0.210 | 0.226 | 1.000 |
| **Th1** |  |  |  |  |  |  |
| IFN-γ | -0.193 | 0.894 | 1.000 | -0.160 | 0.359 | 1.000 |
| IL-12(p40) | -0.165 | 0.301 | 1.000 | -0.170 | 0.329 | 1.000 |
| IL-2 | -0.221 | 0.284 | 1.000 | -0.074 | 0.672 | 1.000 |
| MIG/CXCL9 | 0.133 | 0.279 | 1.000 | -0.253 | 0.143 | 1.000 |
| INF-λ3/IL-28β | 0.187 | 0.384 | 1.000 | -0.249 | 0.150 | 1.000 |
| IP-10/CXCL10 | -0.115 | 0.647 | 1.000 | -0.263 | 0.128 | 1.000 |
| I-TAC/CXCL11 | -0.276 | 0.073 | 0.863 | -0.335 | **0.049** | 1.000 |
| **Th2** |  |  |  |  |  |  |
| IL-4 | 0.196 | 0.408 | 1.000 | 0.023 | 0.896 | 1.000 |
| IL-5 | -0.179 | 0.925 | 1.000 | -0.394 | 0.019 | 0.6755 |
| **Th17** |  |  |  |  |  |  |
| IL-17A | -0.224 | 0.008 | 0.115 | -0.033 | 0.851 | 1.000 |
| IL-17F | -0.105 | 0.715 | 1.000 | NA | NA | NA |
| IL-22 | -0.099 | 0.220 | 0.997 | NA | NA | NA |
| IL-21 | -0.012 | 0.513 | 1.000 | -0.026 | 0.883 | 1.000 |
| IL-23 | 0.022 | 0.948 | 1.000 | NA | NA | NA |
| IL-17E | -0.107 | 0.642 | 1.000 | NA | NA | NA |
| IL-27 | 0.135 | 0.351 | 1.000 | -0.068 | 0.697 | 1.000 |
| **B cell** |  |  |  |  |  |  |
| BCA-1/CXCL13 | -0.195 | 0.475 | 1.000 | -0.543 | 0.007 | 0.245 |
| SDF-1α+β/CXCL12 | -0.282 | 0.228 | 0.999 | 0.010 | 0.954 | 1.000 |
| BAFF | -0.102 | **0.034** | 0.586 | -0.155 | 0.375 | 1.000 |
| APRIL | -0.209 | 0.493 | 1.000 | 0.077 | 0.661 | 1.000 |

^a^ Spearman's Rho-rank based correlation; values>0 indicate positive associations, values <0 indicate negative associations. ^b^ The association was tested using Spearman's method. Statistically significant p values (p<0.05) are in bold. CSF-cerebrospinal fluid. NA-not avaiable due to no variability in concentrations.
